# A longitudinal quantitative study of the effects of training on food handlers’ hygiene knowledge and practices in the National School Nutrition Programme, Western Cape, South Africa

**DOI:** 10.4102/hsag.v31i0.3423

**Published:** 2026-09-23

**Authors:** Hayley K. Langley, Izanne S. Human, James P. Odendaal

**Affiliations:** 1 Department of Environmental and Occupational Studies, Faculty of Applied Sciences, Cape Peninsula University of Technology, Cape Town, South Africa

**Keywords:** food safety, National School Nutrition Programme, food handler, knowledge, practices, intervention, children

## Abstract

**Background:**

The National School Nutrition Programme (NSNP) provides food to approximately 9.7 million learners across South Africa. Children are more vulnerable to foodborne disease, especially if food is prepared unhygienically. Although foodborne disease outbreaks occur in South African schools, few studies have examined the knowledge and practices of food handlers in the NSNP.

**Aim:**

The aim of the study was to determine whether food hygiene knowledge training intervention at schools offering the NSNP will improve food handlers’ knowledge and practices regarding food hygiene and safety.

**Setting:**

The study was conducted at 10 schools within a Cape Winelands sub-district municipality offering the NSNP.

**Methods:**

A quantitative longitudinal design was employed, utilising a pre-test–post-test intervention study. Data were collected using structured questionnaires to assess knowledge, and an observation checklist was used to assess the practices of the food handlers before and after training.

**Results:**

Pre-training findings showed that most food handlers have a high baseline food hygiene knowledge, yet observations found poor compliance with required practices. After the training intervention, no statistically significant differences (*p* < 0.05) were found between pre- and post-training questionnaire results. However, post-training observations found clear improvement in hygiene practices.

**Conclusion:**

Despite improvement, the study revealed that a lack of resources is the main challenge in implementing safe food handling practices in the NSNP.

**Contribution:**

This study underscores the need to strengthen compliance and routine inspections and prioritise food safety within the NSNP. Future studies should explore behavioural and attitude factors that influence these food handlers.

## Introduction

Global school feeding schemes play a crucial role in addressing child hunger, improving nutrition and enhancing educational outcomes across various regions. These initiatives are designed to tackle food insecurity among learners, thereby promoting regular school attendance and better academic performance. The World Food Programme’s (WFP) school feeding programme operates in many low-income countries, providing meals to increase school participation and improve children’s health (WFP 2022).

In South Africa, the National School Nutrition Programme (NSNP) provides daily food for learners in schools.

The programme is funded by a grant from the National Treasury, with meals prepared by volunteer food handlers (South Africa 2026). However, the NSNP faces challenges such as the absence of infrastructure, lack of contractual continuity, resistance from stakeholders, disregard for infrastructure and insufficient training (Mafugu 2021:1982–1989). Foodborne disease outbreaks and deaths can be caused by poor food hygiene practices, and food can become contaminated during any stage of preparation (Madlala 2022). Therefore, it is important for food handlers to receive food hygiene and safety training to ensure safe food handling practices (Madlala 2022).

Estimates made by the World Health Organization (WHO) revealed that 70% of diarrheal diseases in African children are due to contaminated complementary food. In South Africa, such situations lead to 30 660 child deaths per year (WHO 2017). It is known that in South Africa, foodborne illnesses are not properly reported and documented, even though occurrences are common. Food hygiene refers to all conditions and measures to ensure the safety and sustainability of food, which must be maintained throughout all stages. Food hygiene is concerned with the prevention of different illnesses caused by food contamination (Kilungu 2010). Proper handling and food preparation reduce the risk of food contamination and the transmission of foodborne illnesses (Kilungu 2010).

Looking back into the history of food hygiene, most health complications are caused by faulty hygiene practices or poor knowledge of hygiene practices among the kitchen staff.

Approximately 418 million children, including 9.7 million South African learners, are served by feeding schemes (Western Cape Government 2024; WFP 2022). In the Western Cape, 527 234 learners in 1051 institutions benefitted from the NSNP in the 2023/2024 financial year (Western Cape Government 2024). The district municipality investigated in this study has 52 schools, of which at least 45 participate in the NSNP (Western Cape Government 2021). These programmes, while critical for addressing food insecurity, pose a risk for foodborne disease outbreaks. Foodborne diseases cause considerable morbidity and mortality globally, impacting individuals of all ages, with a particularly high burden among populations in low- and middle-income countries, as well as children under the age of five (National Institute of Communicable Diseases 2021:3–5). According to the WHO (2020), not all food handlers fully grasp the significance of adhering to proper hygiene practices during food preparation. It is essential for food handlers to prevent foodborne outbreaks in food outlets and preparation areas (Madlala 2022).

Foodborne disease outbreaks associated with school feeding schemes remain a significant public health concern in South Africa. In 2011, approximately 2560 foodborne illness outbreaks were recorded, with substantial proportions affecting primary and secondary school learners (Sibanyoni, Tshabalala & Tabit 2017:1397–1406). Several incidents since then have reinforced the vulnerability of school feeding programmes to food safety non-compliance. Notably, outbreaks in Gauteng, Limpopo and the North West province resulted in large numbers of learners being hospitalised after consuming food provided through school feeding schemes. The causes were linked to bacterial contamination, poor hygiene practices and inadequate food storage conditions (Devereux et al. 2018). Investigation identified non-compliance with basic food safety requirements and inadequate infrastructure. More recently, in 2023, the Eastern Cape Department of Health confirmed a foodborne disease outbreak at two neighbouring schools which affected 120 learners between the ages of 8 and 15 years (News24 2023).

In light of the increasing reports of food poisoning among school-aged children, there is a need to assess the influence of training on food handlers’ hygiene knowledge and practices in the NSNP to ensure all food handlers adhere to the ‘Five Keys to Safer Food’ (WHO 2006) and Regulation 638 of 2018 (Department of Health 2018) to prevent an outbreak in the NSNP. The objectives of the study were to (1) determine the hygiene knowledge and practices of food handlers involved in the NSNP at a selected-subdistrict of the Cape Winelands District Municipality (CWDM) before receiving training, (2) provide food hygiene and safety training to food handlers involved in the NSNP at a selected sub-district of the CWDM after the first round of questionnaires and observations and (3) determine whether hygiene knowledge and practices of food handlers regarding food hygiene and safety in the NSNP at a selected sub-district of the CWDM improved after having received training. Using multiple data collection tools allows for an innovative investigation by capturing statistical shifts in knowledge alongside the practical realities of food handling observed in the field. By integrating quantitative questionnaire results with observations, the study provides a robust rationale for using multiple data collection tools, as questionnaires alone may not explain why certain hygiene knowledge is practised. For this study, equal weight is given to both questionnaires and observations to ensure a holistic understanding.

## Research methods and design

### Study design

A quantitative longitudinal pre-test–post-test intervention study was used to determine the influence of training on the food hygiene knowledge and practices of the food handlers. The study used multiple data collection techniques. The questionnaires were used to pinpoint changes in food handlers’ hygiene knowledge before and after training. Additionally, observation checklists were used to collect data on hygiene practice compliance of the food handlers at the different schools.

### Study area

One sub-district of the CWDM was purposively selected for the research study due to the ease of logistics. The sub-district includes an area of approximately 4517.4 km^2^. Estimates on the population of the sub-district indicate that it is home to 119 962 residents and 31 000 households as per the 2021 statistics (Department of Social Development 2019; Western Cape Government 2021) and consists of five towns. The sub-district has a total of 52 schools, of which 39 participate in the NSNP.

### Study population and sampling method

Two schools were purposively selected from each of the five towns in the selected sub-district. These 10 schools were selected for logistical ease of data collection, as well as the frequency with which the researcher was able to visit the towns. The target population included 100% of the volunteer food handlers who prepared and served food for learners at the purposively selected schools in the selected sub-district.

Although the food handlers are volunteers, they were employed under a 1-year contractual agreement with the schools. For this study, the same employees remained employed and participated throughout. All food handlers of the 10 schools partook in the study. This resulted in a sample population of 30 food handlers who completed the questionnaires and were exposed to the training sessions.

### Data collection

Data were collected by the researcher from one school per day to maintain consistency in the timing of the research visits. This approach ensured that observations occurred around the same time each day, specifically during the busy period when the food handlers were actively preparing lunch. During the initial visit, the researcher observed the daily food hygiene practices of the food handlers as they prepared lunch for the learners. Following the meal service, the food handlers were requested to complete the pre-training questionnaires.

The training was conducted on a different day, after the initial data collection. The content of the training was in accordance with the guidelines contained in the ‘Five Keys to Safer Food’ (WHO 2006) and Regulation 638 of 2018 (Department of Health 2018). Two weeks after the initial questionnaires were completed, the food handlers convened at a central venue for training conducted by the researcher. Following a 3-month interval, a subsequent round of observations and questionnaires was conducted by the researcher.

The post-training questionnaires had a format consistent with the initial pre-training questionnaire, employing similar questions albeit presented in a different format. The pre- and post-training questionnaires were designed and administered by the researcher. The pre- and post-training questionnaires were in different formats to prevent repeated exposure to the same questions, which can lead to improved results due to memorising the questions.

### Data collection tools

Two questionnaires and an observation checklist were used to collect data for this investigation. To assess the hygiene knowledge and practices of participants prior to receiving training (objective 1), a pre-training questionnaire was developed. The questionnaire consisted of closed-ended questions designed to elicit fixed responses. Specifically, it included inquiries aligned with the principles of the ‘Five Keys to Safer Food’ (WHO 2006) and Regulation 638 of 2018 (Department of Health 2018). The questionnaire consisted of questions addressing topics such as keeping clean, separating raw and cooked foods, storing food at safe temperatures, using safe water and materials, cooking food thoroughly (WHO 2006) and adhering to general requirements stipulated for food handlers according to Regulation 638 of 2018 (Department of Health 2018). Responses were recorded on a 5-point Likert scale, where participants indicated their level of agreement with each statement using options ranging from ‘strongly disagree’ (1) to ‘strongly agree’ (5).

For objectives 1 and 3, an observation checklist consisting of 22 observation areas with binary options (‘Compliant’ or ‘Non-compliant’), developed by the researcher, was employed. During two separate visits, the researcher observed the food handling practices of participants. The aim was to assess whether participants applied their knowledge of food hygiene in practice and how this differed before and after training was received. Observations were conducted during two separate visits. These visits were scheduled to coincide with the busy lunch preparation period. Each observation session lasted approximately 60 min, a duration determined during a pilot study. The observations focused on food hygiene practices, including key areas such as proper use of protective clothing, hygiene and personal hygiene, such as handwashing protocols. To classify school-level compliance, the performance of all food handlers in a school was considered collectively. In schools with multiple food handlers, if 50% or more of the food handlers were observed to be non-compliant or compliant in a particular area, the school was marked as non-compliant or compliant for that area. The approach allowed the study to account for variation in individual practices while maintaining a clear, standardised criterion for school-level compliance. To minimise observation bias, the researcher conducted the observations discreetly, avoiding interaction with participants. Observations were recorded in real-time using the checklist to ensure accuracy and reliability.

After the training session, a post-training questionnaire was designed to assess participants’ understanding and application of hygiene practices (objective 3). The questionnaire featured closed-ended questions designed to prompt specific responses and was structured in accordance with the ‘Five Keys to Safer Food’ (WHO 2006) and Regulation 638 of 2018 (Department of Health 2018). Each question was presented in a multiple-choice format, requiring participants to select the most appropriate answer from the options provided.

Food handlers requested that questionnaires and training be made available in Afrikaans. The translation of questionnaires was done by the researcher, who is proficient in Afrikaans. All questions were read out loud by the researcher to ensure a comprehensive understanding among all respondents.

### Training intervention

The intervention consisted of a structured, standardised training programme designed and presented by the researcher. The intervention was delivered to the same food handlers who completed the pre-training questionnaires and underwent the pre-training observations. Ensuring the same participants attended the training was essential to accurately measure changes in knowledge and practices. The training was conducted 2 weeks after completion of the pre-training questionnaires and observations. The session took place at a central school hall, selected for its accessibility to all participating food handlers and its capacity to accommodate the group comfortably. The session was administered by the researcher, who is proficient in Afrikaans, the language requested by all participants. The content of the training was developed in accordance with the ‘Five Keys to Safer Food’ (WHO 2006) and Regulation 638 of 2018 (South Africa. Department of Health 2018). A visual-based training approach was employed to enhance comprehension and retention. In addition to visual aids, a brief educational video, produced by the WHO, was screened, which lasted 5 min. The video illustrated the core principles of the ‘Five Keys to Safer Food’ (WHO 2015).

Complementing these visual tools, a structured 38-slide PowerPoint presentation aligned with Regulation 638 of 2018 (South Africa. Department of Health 2018), outlining the specific duties of the food handlers.

Despite the comprehensive coverage, the presentation was delivered within 30 min. The entire session, with a question-and-answer session, was efficiently concluded within 1 h. No additional training sessions were provided during the follow-up period to ensure that any changes observed could be attributed to this single, standardised intervention.

### Data analysis

Data from the questionnaires were analysed using descriptive statistics (frequencies and percentages) and inferential statistics (chi-square tests) to compare food hygiene knowledge scores before and after the training intervention. Data analysis was performed using the Statistical Package for Social Sciences (SPSS) software. Statistical support was provided by a statistician from the Cape Peninsula University of Technology.

The Pearson’s chi-square test was used to assess the association between pre- and post-training categorical responses, as the study variables were ordinal in nature. Due to the small sample size and the presence of cells with expected counts less than 5, exact *p*-values were used to improve the accuracy of the statistical analyses.

The Likert scale response categories were collapsed prior to analysis to reduce sparse cell frequency. ‘Strongly agree’ and ‘Agree’ responses were combined into a single ‘Agree’ category, and the same was done for the ‘Disagree’ category. Neutral responses were kept as a separate category. This approach was done due to the sample size to ensure a more reliable chi-square approach.

### Pilot study

The questionnaires and observation checklist were developed specifically for this study. The validity of the content was supported through review by the research supervisors, and a pilot test was conducted prior to data collection to assess reliability, clarity, relevance and alignment of pre- and post-training questions. The pilot study was conducted to refine the research design and ensure the feasibility of data collection. Throughout this pilot study, the researcher assessed the time required to administer the questionnaire and to allocate sufficient resources for data collection. The pilot study also indicated the time allocated for the training session. The pilot study identified any questions that were confusing to participants, allowing for necessary revisions to improve clarity and understanding. For the pre-training questionnaire, one question was rephrased, and two questions were changed due to incorrect temperatures in the questions, and seven questions were removed that did not align with the objective of the study. For the post-training questionnaire, four questions were changed due to the options provided being incorrect, and two questions’ options were rearranged to avoid confusion. Additionally, the effectiveness of the questionnaires in addressing the research questions was evaluated.

### Ethical considerations

Ethical clearance to conduct this study was obtained from the Faculty of Applied Sciences Ethics Committee, Cape Peninsula University of Technology (No. 217089593/04/2023). Data collection permission was obtained from the Department of Basic Education (Reference number: 16375E04C0007EB-20230508). The proposal was approved by the University’s faculty ethics committee (Reference number: 217089593/06/2023). Privacy and anonymity were protected; for example, the identity of participants remained anonymous, and each participant was assigned a unique number to further protect their identity. The research was conducted in compliance with the *Protection of Personal Information Act* (Act 4 of 2013) (South Africa 2013). Study participants participated voluntarily after completing and signing an informed consent form. Participants could withdraw their consent to participate at any time. The questionnaires were completed at the different schools’ food preparation areas where the food handlers were comfortable and familiar with the environment. The questionnaires were handed out and collected by the researcher. The data were captured by the researcher and have been loaded onto the CPUT data repository, eSango, in accordance with the CPUT policy.

## Results

Table 1 presents the distribution of responses to statements focusing on the Five Keys to Safer Food (WHO 2006) and R638 of 2018 (Department of Health 2018).

**TABLE 1:** Pre-training results of respondents based on the ‘Five Keys to Safer Food’ and R638 of 2018 (*N* = 30).

Criteria	Variable	Strongly disagree		Disagree		Neutral		Agree		Strongly agree	
		Frequency	%	Frequency	%	Frequency	%	Frequency	%	Frequency	%
Keep clean	It is important to wash hands before handling food and often during food preparation	0	0.0	0.0	0.0	0.0	0.0	3	10.0	27	90.0
	It is important to wash hands after going to the toilet.	0	0.0	1	3.3	0	0.0	1	3.3	28	93.3
	A wiping cloth can spread organisms.	0	0.0	2	6.7	2	6.7	9	30.0	17	56.7
	Kitchen food preparation surfaces do have to be kept clean.	0	0.0	4	13.3	1	3.3	5	16.7	20	66.7
	Hands must be washed with running water and soap for a minimum of 20 s and also dried with a clean dry towel.	0	0.0	1	3.3	0.0	0.0	3	10.0	26	86.7
Separate raw and cooked food	Raw and cooked food must be stored separately.	0	0.0	2	6.7	0.0	0.0	5	16.7	23	76.7
	Raw food should be stored on the top shelves of the refrigerator.	5	16.7	7	23.3	5	16.7	5	16.7	8	26.7
	Cooked food does not have to be stored on the top shelves of the refrigerator.	10	33.3	7	23.3	6	20.0	3	10.0	4	13.3
Cook food thoroughly	Meat must be properly cooked so that it reaches 40 °C at the core of the thickest part of the meat.	4	13.3	3	10.0	0.0	0.0	12	40.0	11	36.7
	Soup and stews should always be boiled/reheated at 60 °C to ensure safety.	2	6.7	0	0.0	1	3.3	15	50.0	12	40.0
Keep food at safe temperatures	Leftover cooked food must be refrigerated within 2 h of cooking.	6	20.0	2	6.7	6	20.0	2	6.7	14	46.7
	Cooked meat can be left at room temperature overnight to cool before refrigerating.	9	30.0	7	23.3	1	3.3	5	16.7	8	26.7
	Frozen food should be defrosted at room temperature.	1	3.3	6	20.0	2	6.7	10	33.3	11	36.7
	The temperature of a fridge must be at 4 °C.	3	10.0	7	23.3	2	6.7	6	20.0	12	40.0
	The temperature of the freezer must be −12 °C.	0	0.0	6	20.0	6	20.0	7	23.3	11	36.7
Use safe water and raw materials	Food should be checked for freshness and quality.	1	3.3	0	0.0	0	0.0	9	30.0	20	66.7
	Vegetables should first be chopped up and then washed.	8	26.7	9	30.0	0	0.0	4	13.3	9	30.0
	Damaged cans cannot be used.	10	33.3	2	6.7	2	6.7	3	10.0	13	43.3
	Do not use food that has reached its expiration date.	4	13.3	0	0.0	0	0.0	8	26.7	18	60.0
General questions based on R638 of 2018	Refuse bins do have to be covered with a lid.	1	3.3	1	3.3	0	0.0	4	13.3	24	80.0
	Protective clothing may not be worn outside of the preparation areas.	14	46.7	5	16.7	2	6.5	3	10.0	6	20.0
	There is no need to wear protective clothing such as hairnets and gloves other than the work uniform.	2	10.0	1	3.3	1	3.3	2	6.7	23	76.6

Figure 1 illustrates the post-training responses that assessed the respondents’ knowledge based on Key 1 ‘Keep clean’.

**FIGURE 1:**
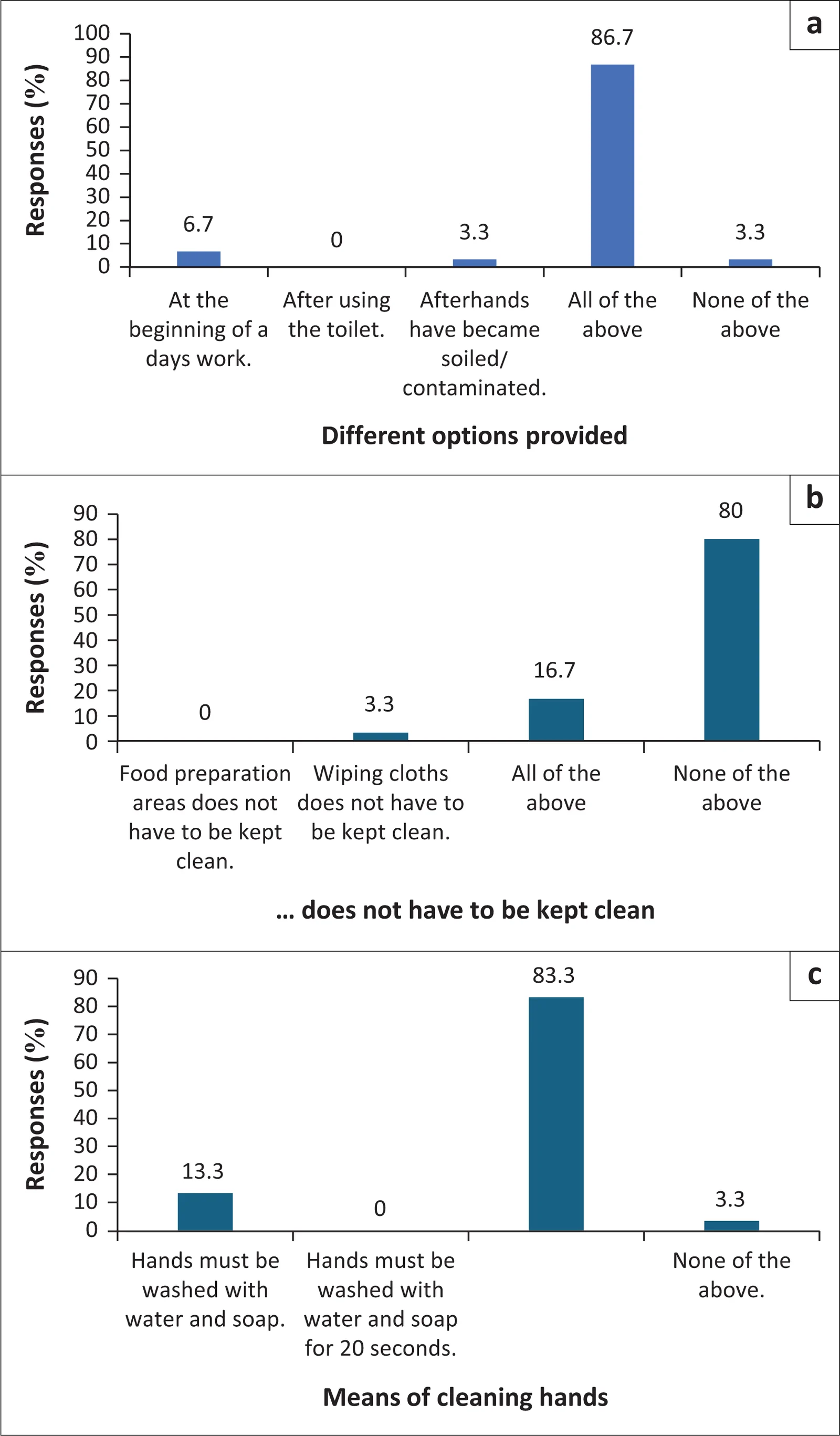
Post-training questionnaire responses to: (a) key moments to wash hands, (b) keeping clean practices and (c) means of cleaning hands (*N* = 30).

Figure 2 illustrates the respondents’ post-training responses based on Key 2 ‘separate raw and cooked food’.

**FIGURE 2:**
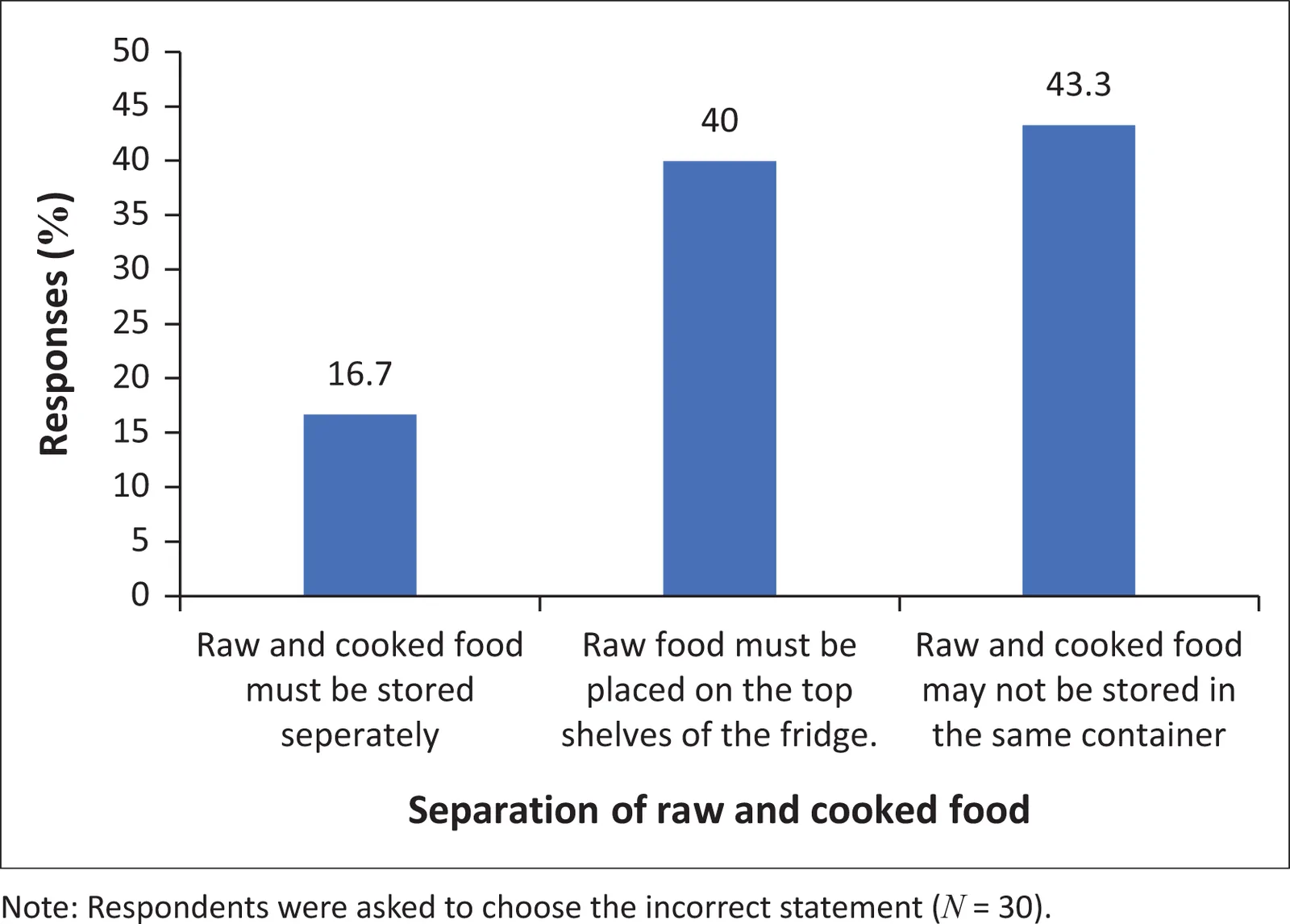
Post-training responses to the question on separating raw and cooked food.

Figure 3 illustrates the respondents’ post-training answers to questions assessing their knowledge based on Key 3 ‘Cook food thoroughly’ and Key 4 ‘Keep food at safe temperatures’.

**FIGURE 3:**
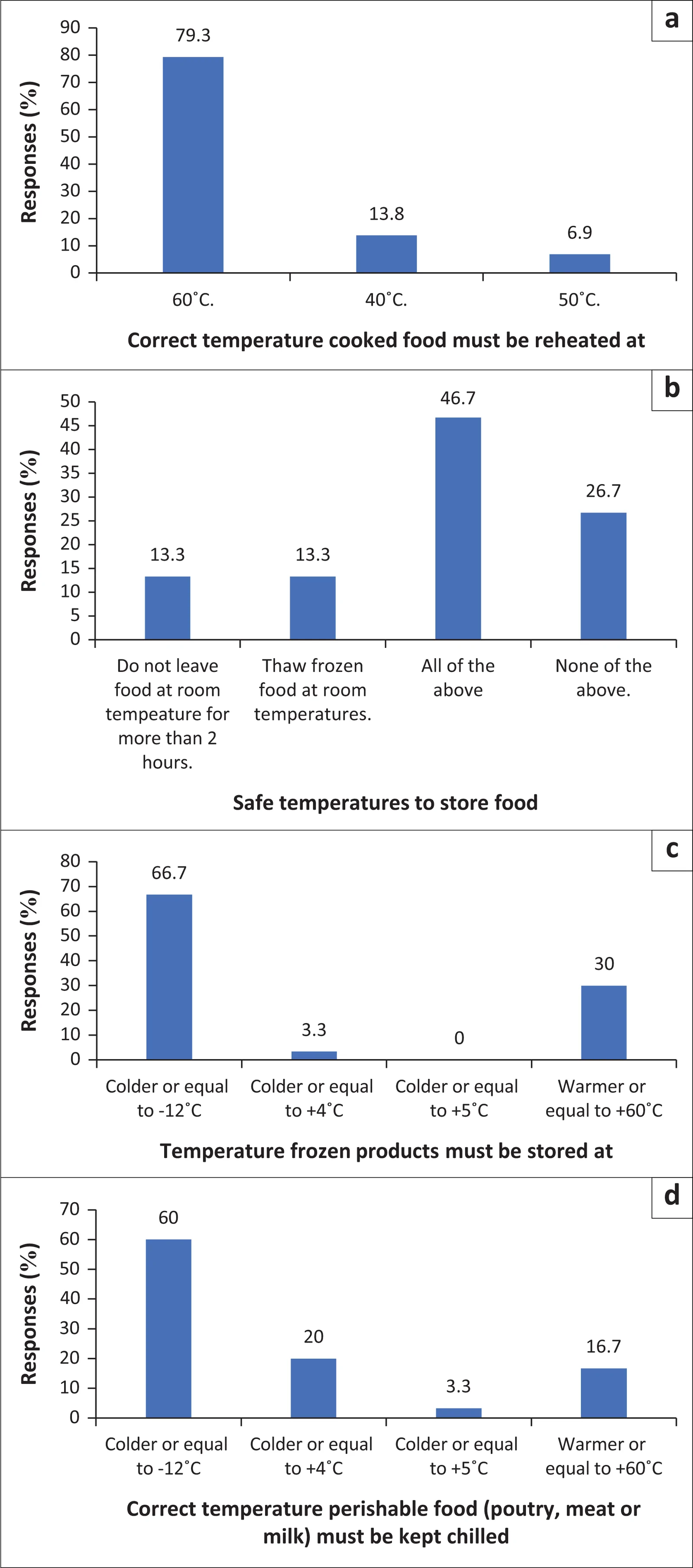
Post-training responses to questions on: (a) correct temperature at which food must be reheated (*N* = 29), (b) safe temperatures to store food (*N* = 30), (c) the temperature frozen products must be stored at (*N* = 30) and (d) the correct temperature perishable food must be kept chilled (*N* = 30).

Figure 4 illustrates the post-training responses based on Key 5 ‘Use safe water and raw materials’ and questions related to R638 of 2018.

**FIGURE 4:**
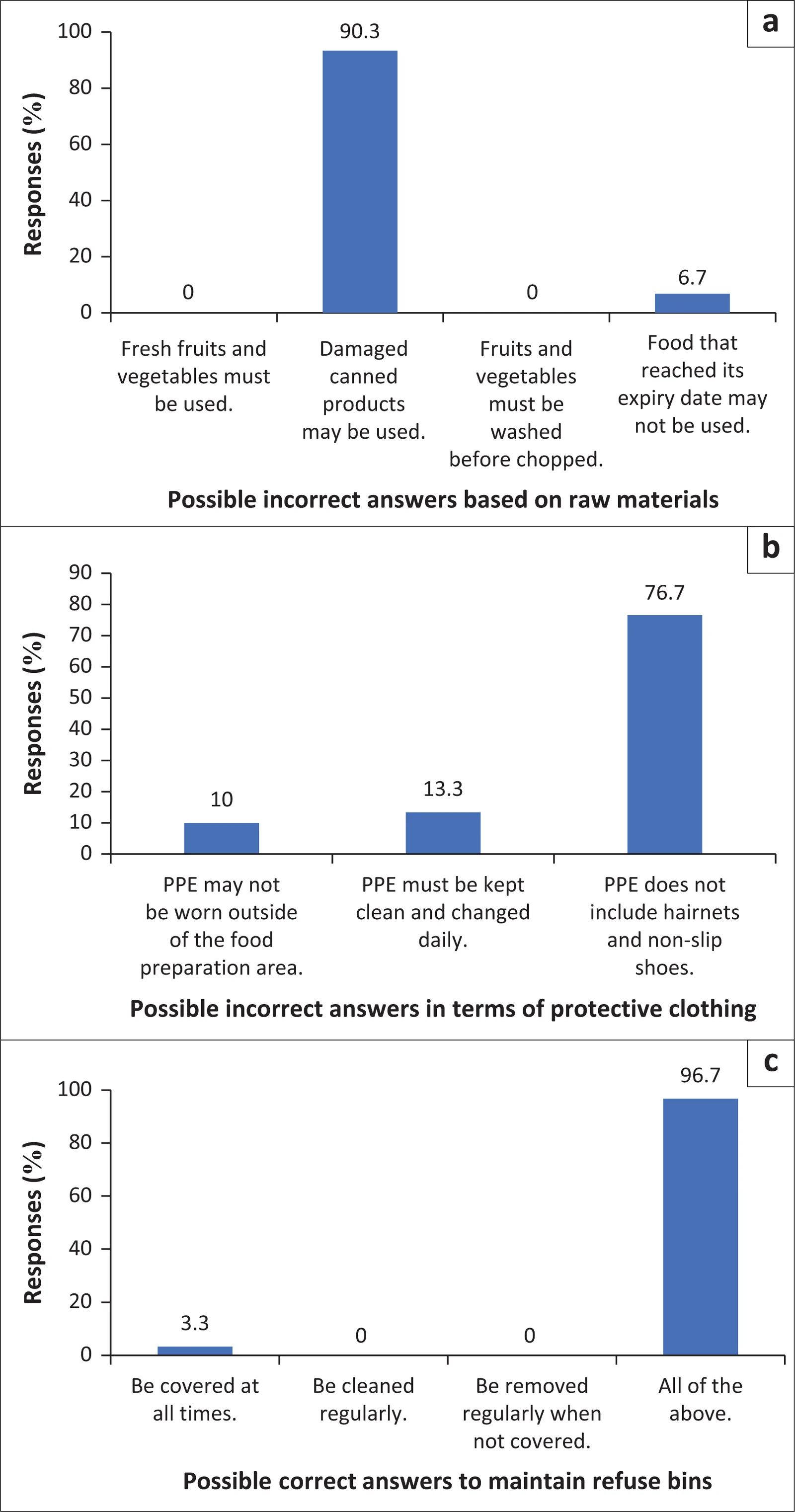
The responses to post-training questions on: (a) possible incorrect statements related to raw materials, (b) possible incorrect answers in terms of protective clothing and (c) possible correct answers in terms of the maintenance of refuse bins in the food preparation area (*N* = 30).

Table 2 shows the pre- and post-training observations made by the researcher during visits at the different schools. Observations were made based on the Five Keys to Safer Food and R638 of 2018.

**TABLE 2:** Pre- and post-training observations made at the 10 selected schools based on the Five keys to safer food and R638 of 2018 (*N* = 10).

Observation	Schools			
	Pre-training		Post-training	
	Compliant	Non-compliant	Compliant	Non-compliant
Hands are washed regularly.	2	8	10	0
Hands are washed with soap and running water for 20 s and dried.	2	8	2	8
Food preparation area is clean.	8	2	10	0
All cloths used are clean.	2	8	9	1
Continuous cleaning is done.	8	2	10	0
Dishes and kitchen equipment are washed.	9	1	10	0
Raw and cooked food are kept separate.	7	3	10	0
Food is cooked to a core temperature of +60 °C or warmer.	10	0	10	0
Food is kept chilled at a temperature of +4 °C or colder.	10	0	10	0
Frozen products are stored at a temperature of −12 °C or colder.	10	0	10	0
Temperatures are displayed/monitored.	0	10	2	8
The thawing process is as short as possible.	10	0	10	0
Safe/clean water is used.	10	0	10	0
Fresh foods are used.	10	0	10	0
Fruits and vegetables are washed before being used or eaten.	0	10	9	1
No products are used that have reached their expiry date.	10	0	10	0
Bins are covered.	0	10	6	4
Correct PPE provided and used correctly.	4	6	9	1
PPE are kept clean.	4	6	10	0

PPE, personal protective equipment.

Table 3 summarises the pre- and post-training Pearson chi-square analysis of responses to identify whether there were any significant changes in the respondents’ knowledge.

**TABLE 3:** Summary of pre- and post-training Pearson chi-square analysis. The significant *p*-value is *p* < 0.05.

Criteria	Topic assesses pre- and post-training	*p*-value	Pearson Chi-square statistical analysis
Key 1: Keep clean	Handwashing importance (before food handling, during preparation and after toilet use)	1	No significant difference
	Wiping cloths must be kept clean.	1	No significant difference
	Means of handwashing.	1	No significant difference
	Keep kitchen surfaces and food preparation areas clean.	0.329	No significant difference
Key 2: Separate raw and cooked food	Storing raw and cooked food in the refrigerator correctly.	< 0.05	Significant difference
	Store raw and cooked food separately.	1	No significant difference
Key 3: Cook food thoroughly	Reheating meat.	0.089	No significant difference
	Reheating soups and stews.	0.167	No significant difference
Key 4: Keep food at safe temperatures	Refrigerating leftover cooked food or storing food at safe temperatures.	0.008	Significant difference
	Store cooked food at safe temperatures.	0.140	No significant difference
	Defrosting food at room temperature.	0.053	No significant difference
	Refrigerate and keep perishable food chilled at +4 °C or colder.	0.122	No significant difference
	Freezer temperatures −12 °C	0.550	No significant difference
Key 5: Use safe water and raw material	Check food for quality and/or freshness.	1	No significant difference
	Using expired food products.	1	No significant difference
	Wash vegetables before chopping.	0.179	No significant difference
	Damaged cans cannot be used.	1	No significant difference
R638/2018	Maintaining refuse bins.	1	No significant difference
	Personal protective clothing cannot be worn outside the food preparation area.	0.194	No significant difference
	Correct personal protective clothing to be worn.	1	No significant difference

## Discussion

### Overall impact of food safety training

The study assessed the impact of food hygiene and safety training on the food handlers in the NSNP. Findings demonstrated limited significant improvement in food handlers’ knowledge after training was received (Table 3). Findings of the observation checklist indicated improvement in several food handling practices (Table 2). This indicates that training positively impacted food handlers’ practices even when measurable changes in their knowledge are limited.

### Baseline food safety knowledge among food handlers

Pre-training results revealed that food handlers had a moderate to high baseline knowledge regarding food hygiene and safety principles before the training intervention was received. A high baseline knowledge was particularly observed (Figure 1) in areas such as ‘Keep clean’. Similar findings have been reported in other food safety studies. In these studies, food handlers displayed acceptable food hygiene knowledge prior to the intervention study (Kirchner et al. 2023:A44–A57). The lack of statistically significant differences seen between pre- and post-training questionnaires may therefore partly be explained by a ceiling effect due to the prior knowledge of participants. Moderate baseline knowledge was also observed in questions assessing ‘separate raw and cooked food’ and ‘use of safe resources and raw materials’.

### Hygiene knowledge of food handlers

Although pre-training results found a moderate to high baseline knowledge, several misconceptions regarding food hygiene knowledge remained evident post-training. The descriptive post-training questionnaire results indicated that uncertainty remained in areas such as ‘separate raw and cooked food’ (Figure 2) and ‘keeping food at safe temperatures’ (Figure 3). Similar results have been found in studies conducted among school food handlers. This was particularly seen regarding food control and cross-contamination prevention (Teffo & Tabit 2020:311; Zelalem et al. 2021:501–509). These findings suggest that food safety concerns that have technical measurements and regulatory requirements may be more difficult to fully understand and apply consistently.

Although some improvement was seen in the descriptive post-training questionnaire results, for ‘keep clean’ (Figure 1), ‘cooking food thoroughly’ (Figure 3(a)), ‘use safe water and raw materials’ (Figure 4(a)) and general questions based on R638 of 2018 (Figure 4(b) and Figure 4(c)) these results were not statistically significant. Due to the lack of statistical improvement, these results must be interpreted with caution. The improvement in the descriptive results of the post-training questionnaire results may not be attributed to the training intervention but could have occurred by chance.

### Hygiene practices of food handlers

Descriptive pre-training observation results showed that in some areas, food handlers had the knowledge but did not practice the knowledge they had, as pre-training observation results found. In contrast with the pre-training questionnaire findings, pre-training observation results improved after training was received (Figure 2). Post-training observation results found improvements in areas such as handwashing frequency, cleaning the food preparation area, separating raw and cooked food and washing fruits and vegetables before chopping, proper refuse bins and use of personal protective clothing. Similar discrepancies between food hygiene knowledge and practices have been reported in previous studies. These studies found that food handlers demonstrated acceptable hygiene practices despite limited formal understanding of food safety principles (Mohamed & Evans 2024:100386; Sheehama & Singh 2025:631). This suggests that a change in hygiene practices may occur even when theoretical knowledge results remained unchanged.

### Discrepancies between knowledge and practices

Several explanations can account for discrepancies between the food handlers’ knowledge and practices. Firstly, the improvement in practices could have been influenced by social desirability bias. Social desirability bias is a form of positive response bias that may influence the questionnaires. This occurs when respondents give the more favourable answer to present themselves positively to others. This type of bias may involve self-deceptive enhancement, where responses are intentionally altered to create a more socially acceptable image (Grower et al. 2022:102159). In this study, results may be due to the food handlers selecting responses they believed were expected rather than reflecting their true knowledge or routine practices. Therefore, social bias can be the reason the knowledge and practices differed.

Secondly, the improvement of the food hygiene practices may have been influenced by the Hawthorne effect. The Hawthorne effect is a psychological phenomenon where individuals adjust their behaviour, often by improving. This is simply because they are aware that they are being observed, rather than actual changes in their environment or practices (Berkhout et al. 2022:1033486). Food handlers may have temporarily improved their behaviour because they are aware that observations were being conducted. Consequently, some of the improvements that were seen may not fully reflect in the routine day-to-day hygiene practices under normal food handling conditions. However, these findings remain important, as these results suggest that food safety and hygiene training, together with supervision and ongoing monitoring, may positively improve food handlers’ hygiene practices within the NSNP. This further highlights the potential value of regular supervision and accountability measures in promoting food safety compliance among the food handlers in the NSNP.

### Structural and environmental barriers to food safety compliance

Post-training observation results also revealed that in some areas, such as washing hands with soap and water, temperature monitoring devices and availability of refuse bins, no improvements were found. For example, post-training observations showed an improvement in the frequency of handwashing among food handlers; however, they did not use soap because it was not available. Food handlers also did not have access to temperature monitoring devices and waste bins. Although training was received, most of the schools still lacked these essential tools after training. A study conducted by Chakma et al. (2024:e272886), found that the effectiveness of training may be limited by factors such as the availability of resources. The study found that infrastructure is a prerequisite for behaviour change (Chakma et al. 2024:e27286). Structural and environmental limitations within the participating schools may have influenced the relationships between hygiene knowledge and practices. These limitations may restrict food handlers’ ability to implement the recommended food safety practices they were taught during the training interventions.

### Implications for National School Nutrition Programme food safety programmes

The findings of this study have important implications for the implementation of food safety programmes within the NSNP. Food safety training alone may be insufficient to achieve sustained improvements in food handling practices without ongoing monitoring, refresher training, managerial support and the provision of adequate hygiene resources. Similar findings were reported by Insfran-Rivarola et al. (2020:1169), who found that although training improved food handlers’ knowledge and practices, sustained behavioural change often required practical training approaches, continuous reinforcement and ongoing supervision. The findings of the present study, therefore, suggest that future NSNP food safety interventions should combine theoretical training with practical demonstrations, regular supervision and improved access to hygiene resources such as soap, temperature-monitoring devices and protective clothing to strengthen long-term food safety compliance.

### Limitations

This study had several limitations that must be considered. Firstly, the small sample population of *N* = 30 food handlers limited the statistical power of the study. This may have reduced the ability to identify statistically significant differences between pre- and post-training questionnaire results. The Likert scale used also had multiple response categories that may have affected the robustness of the statistical analysis due to the low expected cell counts in certain categories. Secondly, the observational aspects of the study may have been influenced by the Hawthorne effect. The effect occurs when food handlers temporarily improve their behaviour or practices because they are aware of observations being conducted. Thirdly, the use of self-reported questionnaires may also have introduced social desirability bias, as respondents may have selected responses that they believed were expected of them. The training intervention had a relatively short follow-up period of 3 months. This may have limited the ability to assess the impact of the intervention. The 3-month follow-up period was selected in alignment with the national environmental norms and standards for monitoring high-risk food premises by Environmental Health Practitioners (South Africa. Department of Health 2025). A longer follow-up period may have provided a better understanding of the long-term effectiveness and sustainability of training interventions. Lastly, the absence of a control group limited the ability to determine whether changes solely contributed to the changes in practices.

### Recommendations

Many limitations were linked to the lack of basic supplies such as soap, water, paper towels or thermometers. Provision of sufficient resources should accompany training to improve the effectiveness of training. Relevant authorities such as the Department of Basic Education should address structural and equipment challenges. Local authorities, including Environmental Health Practitioners, must ensure that regular monitoring and training of the food handlers in the NSNP is prioritised to ensure compliance. Given that many food handlers demonstrated improved practices despite no statistically significant differences in knowledge, future studies can include and investigate behaviour change strategies or the attitude of food handlers.

The findings of this study are limited to food handlers participating in the NSNP within a Cape Winelands sub-district municipality and therefore cannot be generalised to other settings or populations.

## Conclusion

The findings demonstrated that while the training intervention did not show statistically significant improvement in most of the knowledge areas, improvements were seen in the actual hygiene practices throughout all the schools. These results are similar to the literature suggesting that the behavioural or practice observations may provide a more accurate indication of the effectiveness of training than a written assessment (Da Cunha 2019:108637). Pre-training descriptive results revealed a high baseline knowledge among the food handlers. Food handlers had knowledge of hand hygiene practices and the importance of keeping the food preparation area clean. However, uncertainty or misconceptions were observed particularly regarding keeping food at safe temperatures and cooking food thoroughly. Post-training observations found positive changes in the practices of food handlers such as separating raw and cooked food, keeping food at safe temperatures, cooking food thoroughly, keeping the food preparation area and equipment clean and making use of clean and safe water and raw materials. While the post-training questionnaire results did not show a statistically significant difference compared to the pre-training questionnaire, the food hygiene practices improved in areas such as keeping clean, separating raw and cooked food and using clean and safe water and raw materials. Improvements in practices were more evident than improvements in theoretical knowledge.
